# Fatty acid binding protein 5 promotes metastatic potential of triple negative breast cancer cells through enhancing epidermal growth factor receptor stability

**DOI:** 10.18632/oncotarget.3442

**Published:** 2015-01-31

**Authors:** Catherine A. Powell, Mohd W. Nasser, Helong Zhao, Jacob C. Wochna, Xiaoli Zhang, Charles Shapiro, Konstantin Shilo, Ramesh K. Ganju

**Affiliations:** ^1^ Department of Pathology, The Ohio State University, Columbus, OH, USA; ^2^ Center for Biostatistics, The Ohio State University, Columbus, OH, USA; ^3^ Tisch Cancer Institute, Mount Sinai School of Medicine, New York, USA

**Keywords:** Fatty acid binding protein 5, triple negative breast cancer, epidermal growth factor receptor, protein degradation, metastasis

## Abstract

Fatty acid binding protein 5 (FABP5), an intracellular lipid binding protein, has been shown to play a role in various cancers, including breast cancer. However, FABP5 and its role in triple negative breast cancer (TNBC) have not been studied. We show FABP5 protein expression correlates with TNBC, high grade tumors, and worse disease-free survival in a tissue microarray containing 423 breast cancer patient samples. High FABP5 expression significantly correlates with epidermal growth factor receptor (EGFR) expression in these samples. Decreased tumor growth and lung metastasis were observed in FABP5^−/−^ mice othotopically injected with murine breast cancer cells. FABP5 loss in TNBC tumor cells inhibited motility and invasion. Mechanistic studies revealed that FABP5 knockdown in TNBC cells results in decreased EGFR expression and FABP5 is important for EGF-induced metastatic signaling. Loss of FABP5 leads to proteasomal targeting of EGFR. Our studies show that FABP5 has a role in both host and tumor cell during breast cancer progression. These findings suggest that FABP5 mediates its enhanced effect on TNBC metastasis, in part, through EGFR, by inhibiting EGFR proteasomal degradation. These studies show, for the first time, a correlation between FABP5 and EGFR in enhancing TNBC metastasis through a novel mechanism.

## INTRODUCTION

Fatty acid binding protein 5 (FABP5) is a member of the intracellular lipid binding protein family that bind long chain fatty acids and transport them throughout the cell. FABP5 (also epidermal FABP, keratinocyte FABP, psoriasis-associated FABP, and mal1), was first identified in psoriatic lesions, and further characterized in the epidermis [1]. It plays a role in lipid signaling by binding to and shuttling long chain fatty acids and other hydrophobic ligands through the cytoplasm. Through its mediation of lipid signaling, FABP5 has been shown to play a role in inflammatory and metabolic diseases including psoriasis, insulin resistance, obesity, and atherosclerosis [2-6].

FABP5 has been shown to be involved in a variety of cancers including colon, prostate, and breast cancer [7-9]. Previously, FABP5 mRNA was found to be overexpressed in metastatic breast and prostate cancer cell lines compared to non-metastatic cell lines [9]. FABP5 has been shown to play a role in HER2, a member of the epidermal growth factor receptor family, tumorigenesis [10]. However, an association of FABP5 with epidermal growth factor receptor 1 (EGFR) has not been reported. FABP5 has been previously identified as being pro-tumorigenic in retinoic acid signaling in triple negative breast cancer (TNBC) patients [11]. Although it was shown that FABP5 mRNA is expressed in TNBC patient samples and predicts worse prognosis, a detailed analysis of its protein expression and especially its correlation with EGFR has not been extensively studied in breast cancer, especially TNBC.

TNBC is an aggressive breast cancer characterized by the absence of estrogen receptor (ER) and progesterone receptor (PR) as well as a lack of over-expression of the HER2/Neu receptor. TNBC accounts for ~15% of all breast cancer cases and lacks effective therapeutic strategies. Though primary tumors do respond to chemotherapy; disease-free and overall survival are less than patients with non-TNBC [12]. TNBC is especially challenging, as endocrine therapies are ineffective due to the nature of the cancer. Additionally, there are no current targeted therapies available to patients with TNBC. TNBC is also more likely to metastasize and metastasis is a major contributor in patient mortality [13-16]. EGFR is overexpressed, but not mutated, in TNBC and has been associated with worse prognosis in TNBC patients [17-19]. EGFR targeted therapies have yet to be beneficial in clinic, however, pre-clinical studies continue to develop anti-EGFR therapies [20-22]. Current therapeutic options for TNBC are limited and so far, not effective in inhibiting metastasis, therefore further understanding of the disease is needed.

In the present study, we did a detailed analysis of FABP5 expression, its prognostic value, and its correlation with EGFR in a large cohort of patient samples. Using FABP5 knockout mice and FABP5 down-regulated TNBC cell lines, we studied the functional and mechanistic role of FABP5 in TNBC metastasis. These findings suggest a novel role for FABP5 regulating EGFR in TNBC and have potential for developing targeted therapies against FABP5 for metastatic TNBC.

## RESULTS

### FABP5 is expressed in triple negative breast cancer and associates with worse prognosis

**Figure 1:**
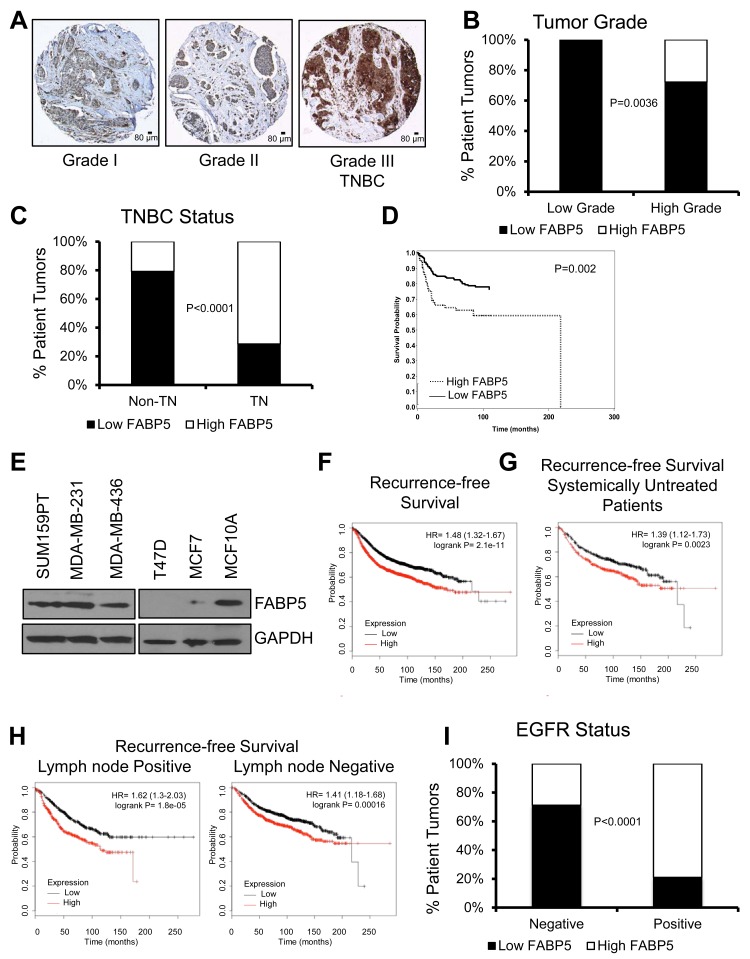
FABP5 expression correlates with worse prognosis in patient samples (A) Representative images from SFBCAS tissue microarray immunohistochemically stained for FABP5, scale bar is 80 μm. (B) Graphical representation of FABP5 expression in patient samples with low and high grade tumors. (C) Graphical representation of FABP5 expression in patient samples with TNBC status. (D) Disease-free survival of patients expressing low or high FABP5. (E) Western blot analysis of TNBC cell lines MDA-MB-231, MDA-MB-436, and SUM159PT, ER^+^ cell lines, T47D and MCF7, and an epithelial cell line, MCF10A, for FABP5 expression (15 kDa) with GAPDH (37 kDa) as a loading control. (F) Recurrence-free survival analysis from 3,455 patients expressing high or low FABP5 (202345_s_at) dichotomized by median FABP5 expression. (G) Recurrence-free survival in 1,000 systemically untreated patients expressing high or low FABP5 (202345_s_at) dichotomized by median FABP5 expression. (H) Recurrence-free survival analysis from 936 patients with lymph node positive (left panel) or lymph node negative status (right panel) expressing high or low FABP5 (202345_s_at) dichotomized by median FABP5 expression. (I) Graphical representation of the correlation between FABP5 and EGFR expression in the SFBCAS TMA.

We analyzed FABP5 expression in a tissue microarray (TMA) constructed of 423 primary breast cancer patient samples. We observed high FABP5 protein expression significantly correlates with high grade tumors, TNBC status (Fig. 1A-C, P=0.0036 and P<0.0001, respectively) and worse disease-free survival in our TMA (Fig. 1D, P=0.002). High FABP5 expression is significantly correlated with negative PR, and negative ER staining, and although FABP5 expression associates with HER2 status, there is not an evident trend of FABP5 staining and HER2 expression (Supplemental Fig. S1A-C, P<0.0001, P<0.0001, and P=0.016, respectively). Analysis of three TNBC cell lines, SUM159PT, MDA-MB-231, and MDA-MB-436 revealed basal FABP5 protein expression similar to non-transformed epithelial cell line, MCF10A, compared to low or no FABP5 expression in ER^+^ cells, T47D and MCF7 (Fig. 1E and Supplemental Fig. S1D). Comparison of our TMA findings with larger, publicly available, compiled datasets on KM Plotter [23], showed that FABP5 expression is significantly correlated with worse recurrence-free survival (Fig. 1F). In a cohort of systemically untreated patients, high FABP5 expression predicts worse recurrence-free survival (Fig. 1G). High FABP5 expression correlates with worse recurrence-free survival in patients with and without lymph node metastasis (Fig. 1H, left and right panel, respectively).

Since EGFR is highly expressed in TNBC breast cancer [24] and EGFR has been associated with metastasis and mortality of TNBC [18], we analyzed a potential correlation of EGFR expression in our SFBCAS TMA with FABP5 expression. We found that EGFR status correlates with high FABP5 expression (Fig. 1I, P<0.0001).

### Host FABP5 modulates tumor growth and metastasis

**Figure 2:**
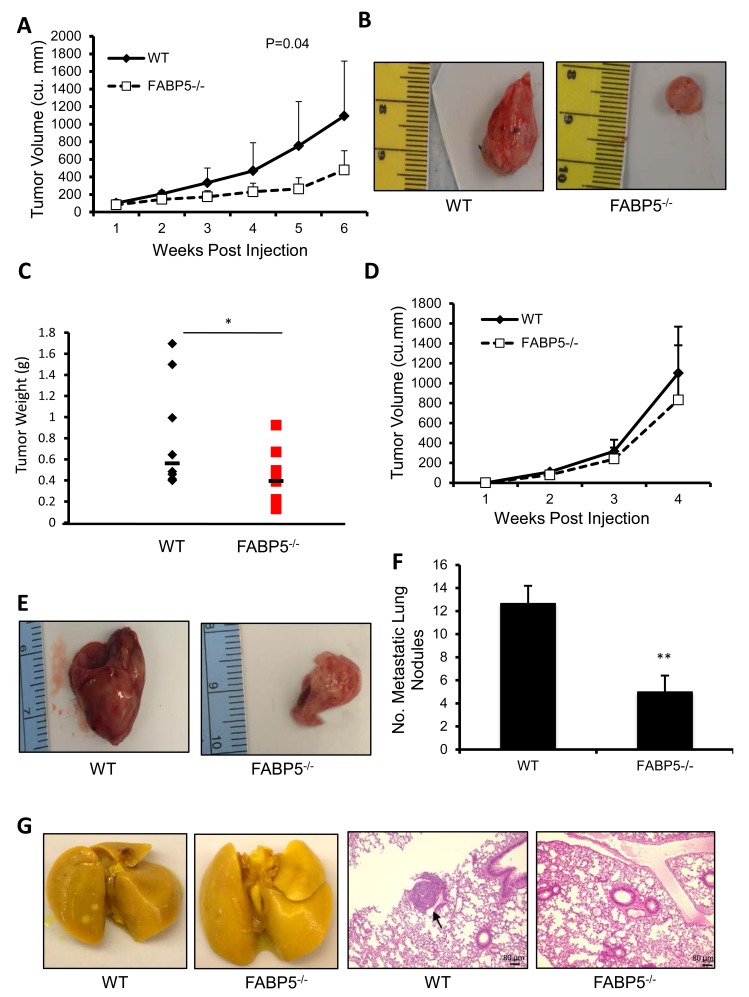
Host FABP5 modulates primary tumor growth and metastasis (A) Tumor growth of PyMT tumors in wild-type (WT) or FABP5 knockout (FABP5^−/−^) mice. (B) Representative images of excised tumors in wild-type (right panel) and FABP5^−/−^ (left panel) mice (n=8/group). (C) Endpoint weights of wild-type and FABP5^−/−^ tumors 6 weeks post injection. (D) Tumor growth of E0771 tumors in wild-type of FABP5^−/−^ mice. (E) Representative pictures of E0771 tumors in wild-type and FABP5^−/−^ mice (lower panel) (n=4/group). (F) Number of metastatic nodules in wild-type and FABP5^−/−^ mice lungs (G) Representative images of whole lungs fixed in Bouin's stain of wild-type and FABP5^−/−^ mice (left panel) and H&E sections of wild-type and FABP5^−/−^ lungs with micro-metastasis (black arrow), scale bar is 80 μm (right panel). Data is represented as mean ± SD.

The patient data supports the role of FABP5 in breast cancer and TNBC. However, this does not address whether FABP5 in the cancer cell, the environment, or both is the contributing factor for worse prognosis in patient data. To address the role of FABP5 in the host, we used an orthotopic injection model in wild-type (WT) and FABP5 knockout (FABP5^−/−^) mice. FABP5^−/−^ mice orthotopically injected with PyMT cells exhibited significantly less tumor volume (P=0.04), significantly slower tumor growth (P=0.035) and smaller tumor weights (P<0.05) compared to wild-type controls (Fig. 2A-C). Next, we employed the metastatic murine breast cancer cell line E0771 to determine if host FABP5 plays a role in distant metastasis [25]. FABP5^−/−^ mice had a slight decrease in tumor growth compared to WT control (Fig. 2D & E). FABP5^−/−^ mice developed significantly fewer metastatic nodules in the lung (Fig. 2F, P<0.01). Micrometastasis was observed through H&E staining in wildtype lungs but none were observed in FABP5^−/−^ mouse lungs (Fig. 2G).

### FABP5 affects cell motility, migration and attachment in triple negative breast cancer cells

**Figure 3:**
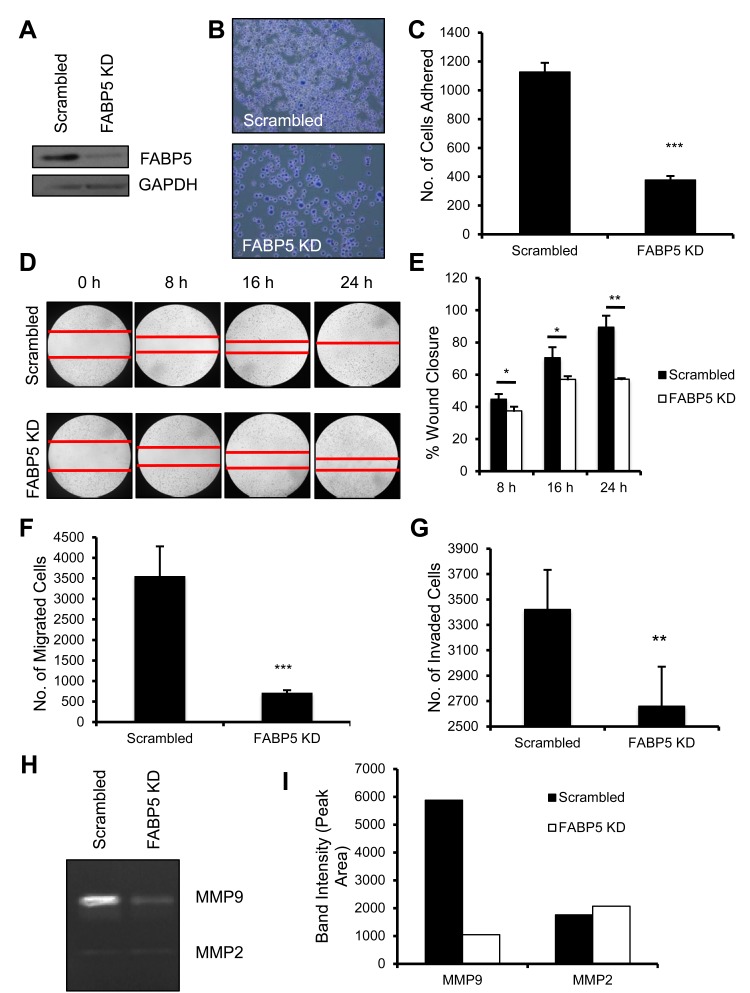
Functional consequences of FABP5 knockdown in TNBC cancer cells (A) FABP5 western blot analysis of shRNA mediated FABP5 knockdown (FABP5 KD) in MDA-MB-231 cells and GAPDH as loading control. (B) Representative image of scrambled control (scrambled) and FABP5 KD cells attached to fibronectin coated wells. (C) Number of scrambled and FABP5 KD cells attached to fibronectin coated wells. (D) Representative images of wound healing of scrambled and FABP5 KD cells in 5% FBS/DMEM over 24 hours. (E) Percentage wound closure of scrambled and FABP5 KD cells over 24 hours. (F) Number of migrated scrambled and FABP5 KD cells after 4 hours in the presence of 5% FBS in DMEM. (G) Number of invaded scrambled and FABP5 KD cells after 24 hours in the presence of 5% FBS in DMEM. (H) Scrambled and FABP5 KD cells were serum starved for 36 hours and media was collected and run on a gelatin zymogram. Gel was stained and imaged for MMP9 and MMP2. (I) Graphical representation of zymogram band intensity of MMP9 and MMP2. Data is represented as mean ± SD.

We have established host FABP5 as necessary in primary tumor development and metastasis. In order to fully grasp the scope of FABP5 in breast cancer, we next studied its role in the cancer cell. In (Fig. 3A), we established a stable shRNA FABP5 knockdown (FABP5 KD) MDA-MB-231 cell line to test the necessity of FABP5 in breast cancer. In order for breast cancer cells to metastasize to distant sites, cancer cells must possess the ability to exit the primary tumor, travel and colonize in a distant tissue [26]. Cell attachment in the metastatic process is imperative for the cells to exit the primary tumor and interact with the extracellular matrix [27]. Using a fibronectin coated plate, FABP5 KD cells and control (scrambled) cells were tested for the ability to attach in serum-free conditions. Fewer FABP5 KD cells attached to the fibronectin surface compared to scrambled control cells (Fig. 3B & C, P<0.001). We tested the functional effect of FABP5 knockdown on the migratory ability of TNBC cells. Wound healing was used to assay cell motility; FABP5 KD cells were significantly slower at wound closure than scrambled cells (Fig. 3D & E, P<0.05 for 8 and 16 hours, P<0.01 for 24 hours). FABP5 knockdown significantly inhibited the ability of the cells to migrate compared to scrambled control (Fig. 3F, P<0.001). Taken together, FABP5 knockdown in TNBC cell line significantly decreases the cell attachment and motility of cells.

We next tested the role of FABP5 in invasiveness of TNBC cells. FABP5 KD cells were less able to invade through a basement membrane invasion assay compared to scrambled control (Fig. 3G, P<0.01). Matrix metalloproteinases are enzymes that degrade the basement membrane and promote invasion and metastasis in cancers [28,29]. As FABP5 KD cell invasion is inhibited, we assayed secretion of MMP9 in supernatants of FAPB5 KD and scrambled control through zymography. FABP5 KD cells secrete less MMP9 compared to scrambled control, however MMP2 secretion was not affected (Fig. 3H & I). Taken together, FABP5, within the cancer cell, is necessary for metastatic potential of TNBC cells.

### FABP5 correlates with EGFR expression and affects EGF-induced motility

**Figure 4:**
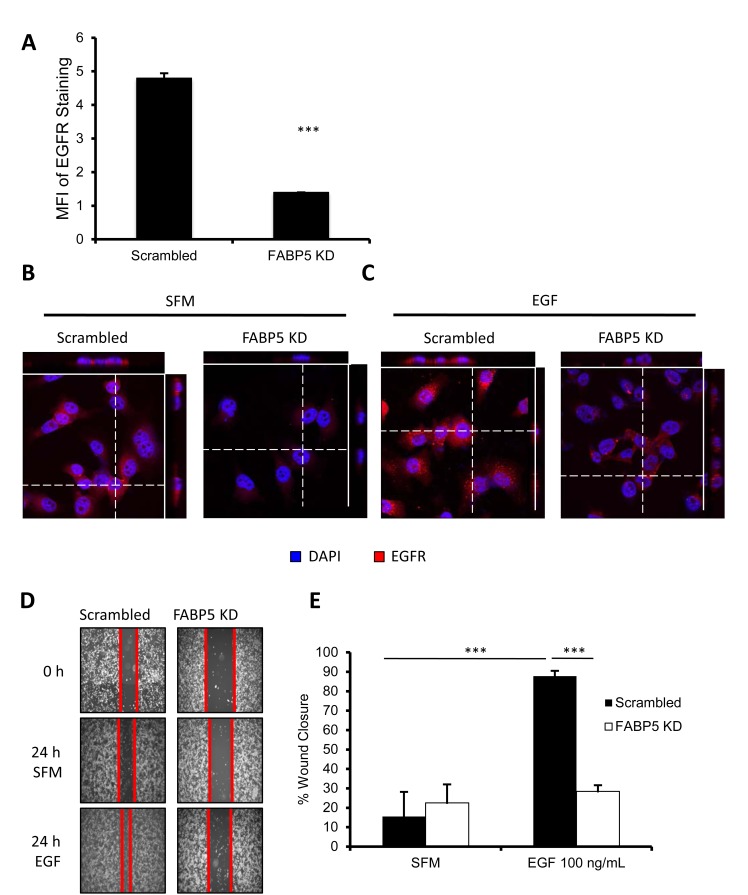
FABP5 mediates metastatic function of TNBC cell line through EGFR (A) Mean fluorescence intensity of EGFR on scrambled and FABP5 KD cells by FACS analysis. (B & C) Confocal microscopy of scrambled and FABP5 KD cells for EGFR (red) expression; DAPI (blue) in serum-free medium (SFM) (B) or EGF (100 ng/mL) stimulation (C). (D) Representative images of scrambled and FABP5 KD wound healing in the absence or presence of EGF (50 ng/mL) at 24 hours. (E) Percentage wound healing of scrambled and FABP5 KD in the absence or presence of EGF (50 ng/mL) at 24 hours. Data is represented as mean ± SD.

Since we observed a correlation between FABP5 and EGFR in breast cancer patient samples, we further analyzed the functional and mechanistic relationship between FABP5 and EGFR in TNBC cells. EGFR expression was reduced in FABP5 KD cells compared to scrambled control cells as analyzed by flow cytometry (Fig. 4A, P<0.001). We further confirmed the decrease of EGFR in FABP5 KD cells through confocal staining (Fig. 4B). We next tested the functional effect of EGFR activation in FABP5 KD and scrambled cells. In (Fig. 4C), EGFR, imaged by confocal microscopy, can be observed in scrambled cells stimulated with EGF, however that effect is lost in FABP5 KD cells. EGF stimulation promoted wound healing in control cells, however, in FABP5 KD cells, wound healing did not respond to EGF stimulation (Fig. 4D & E, P<0.001). Knockdown of FABP5 in TNBC cells decreases EGFR expression and EGF-induced motility of these cells supporting the correlation found in the tissue microarray.

### FABP5 modulates EGFR signaling involved in metastasis in TNBC cells

**Figure 5:**
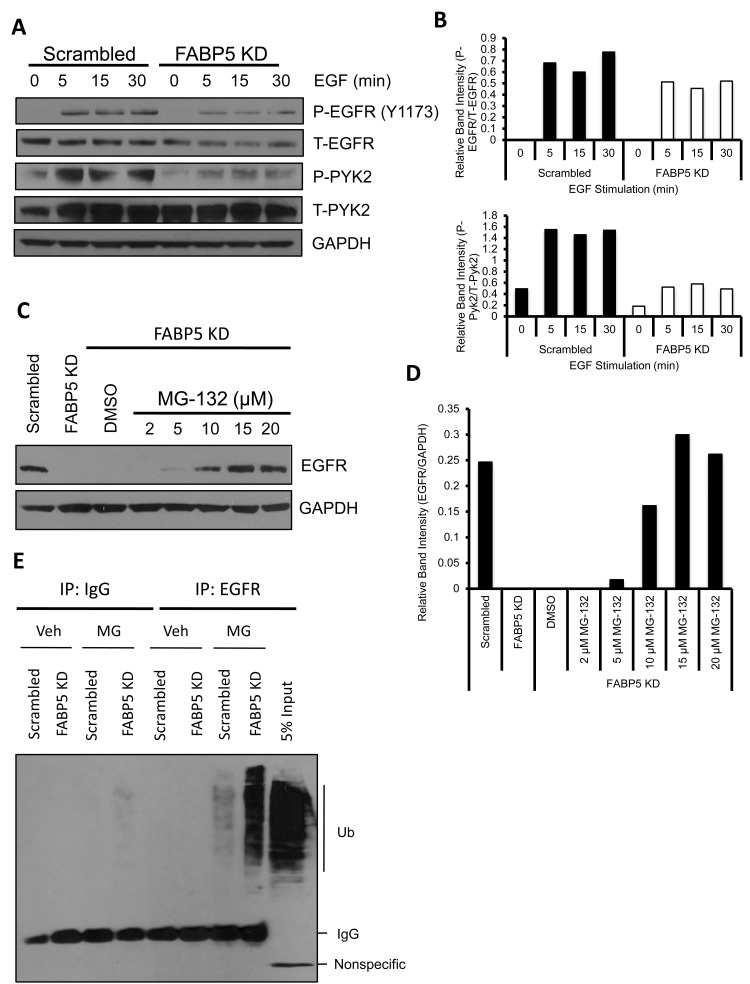
FABP5 KD dampens EGFR mediated downstream signaling of metastatic targets (A) Scrambled and FABP5 KD cells were serum starved overnight and stimulated with EGF (50 ng/mL) for indicated time points. Cell lysates were harvested and subjected to western blot analysis for phospho-EGFR (Y1173), EGFR, phospho-Pyk2, Pyk2, and GAPDH as a loading control. (B) Quantification of relative phospho-EGFR expression in scrambled cells and FABP5 KD cells treated with EGF (50 ng/mL) for varying time pointscalculated with respect to total EGFR expression (upper panel) and relative phospho-Pyk2 expression calculated with respect to total Pyk2 expression (lower panel). C) FABP5 KD cells were treated with varying concentrations of MG-132 or veh for 4 hours. Cells were lysed and analyzed by western blot for EGFR and GAPDH as a loading control. (D) Quantification of relative EGFR expression calculated with respect to GAPDH. (E) Ubiquitin assay of EGFR in scrambled and FABP5 KD cells treated for 4 hours with veh or MG-132 (MG) (15 μM).

Since EGFR expression and EGF-induced functional effects were reduced in FABP5 KD cells, we analyzed the EGFR signaling pathway. In (Fig. 5A & B), FABP5 KD cells exhibit decreased phospho-EGFR (Y1173) and total EGFR expression. Additionally, phospho-Pyk2 was greatly decreased in FABP5 KD cells compared to scrambled cells (Fig. 5A & B). Proline-rich tyrosine kinase 2 (Pyk2), a focal adhesion kinase related protein, mediates cell motility, adhesion and metastasis in various cancers [30]. In order to determine whether phospho-EGFR (Y1173) was the only phospohrylation site affected by FABP5 KD, EGFR phosphorylation sites Y992, Y1045, and Y1068 were analyzed and all sites exhibited decreased phosporylation in FABP5 KD cells compared to scrambled control (Supplemental Fig. S2).

### Loss of FABP5 leads to proteasomal degradation of EGFR

Since we observed a correlation between FABP5 and EGFR in patient samples, we wanted to study the mechanism by which FABP5 regulates EGFR expression. We asked whether EGFR protein is being degraded via the proteasome. MG-132, a proteasome inhibitor was used to test if EGFR was being degraded through proteasomal degradation. In (Fig. 5C & D), we observed that EGFR protein expression increased in FABP5 KD cells after a 4 hour treatment of varying concentrations of MG-132. This increase in protein expression suggests that EGFR is being targeted for proteasomal degradation in FABP5 KD cells compared to scrambled control. To further confirm this finding, an ubiquitin assay was performed to determine the ubiquitination state of EGFR in FABP5 KD cells. EGFR ubiquitination with proteasome blockage was greatly increased in FABP5 knockdown cells compared to scrambled control cells (Fig. 5E). Taken together, knocking down FABP5 expression decreases EGFR expression through targeting EGFR to protein degradation.

## DISCUSSION

TNBC, an aggressive subtype of breast cancer, affects ~15% of all breast cancer patients and is associated with early metastasis and poor prognosis. Treatment of TNBC metastasis proves to be extremely challenging in clinic. Poor prognosis of patients with TNBC is due to the rates of relapse and metastasis [13, 14, 16]. Currently, targeted therapies are not very effective in treating TNBC once it metastasizes. In our studies, we show FABP5 is an excellent biomarker for breast cancer in a TMA containing a large cohort of 423 patient samples. FABP5 protein expression is highly expressed in high tumor grades as well as TNBC samples, which has not been previously shown. These novel findings documented at the protein expression level are supported by previously reported data from gene microarray analysis where such association is evident at the transcriptional level [11].

TNBC metastasis occurs early in patients and is associated with high mortality [15]. There is a lack of effective therapies targeting TNBC metastasis in clinic. In our studies, we show FABP5 in the TNBC tumor cell as well as the host is important for metastasis of TNBC. We show FABP5 promotes motility and invasion of TNBC cells. FABP5 was first identified in psoriatic lesions, was linked to metastasis in 2000 where FABP5 mRNA was expressed in metastatic breast and prostate cancer cell lines compared to non-metastatic cell lines [9]. We showed a role for FABP5 modulation of MMP9 secretion, and not MMP2, in TNBC cells, which plays an important role in degrading the extracellular matrix and the invasion of tumor cells. Similarly, FABP5 was shown to regulate MMP9 and have no effect on MMP2 in oral squamous cell carcinoma [31]. FABP5 in the breast tumor environment is also important in breast cancer progression and metastasis. We showed FABP5 plays a role in both breast cancer growth and metastasis. This role of host FABP5 promoting breast cancer has been previously reported [10,32].

FABP5 plays a dual role, in the tumor cell and the host, in breast cancer tumor growth and metastasis. We showed FABP5 in the tumor micronenvironment is important for tumor growth and metastasis. It has been previously shown in a Her2 overexpressing mouse model that loss of FABP5 decreases murine mammary tumor growth, in part due to loss of host FABP5 [10]. In an inflammatory disease model, athersclerosis, mice deficient for macrophage FABP5 had smaller atherosclerotic legions compared to control and macrophages deficient in FABP5 expressed anti-inflammatory cytokines suggesting a possible role for macrophage FABP5 in inflammatory diseases such as breast cancer [4]. Furthermore, macrophage FABP5 has also been shown to be important in metabolic disease where FABP5 in the macrophage and the adipocyte play an important role in inflammatory signaling [33]. Taken together, this suggests a role for FABP5 in the macrophage during breast cancer progression and metastasis.

It has been previously reported that EGFR overexpression in TNBC correlates with metastasis and worse prognosis for patients [15,17-19]. FABP5 has been shown to play a role in breast cancer in an EGFR family member, HER2, however, not EGFR [10,34]. Our study documents, for the first time, an association between high FABP5 expression and EGFR expression in human breast cancer samples and a novel mechanism by which FABP5 regulates EGFR expression by preventing EGFR proteasomal degradation where the loss of FABP5 targets EGFR for proteasomal degradation. Furthermore, EGF-induced phosphorylation of Pyk2 is dependent, in part, on presence of FABP5. Pyk2 has been previously shown to be phosphorylated by EGFR and Pyk2 has been described to decrease tumorigenesis and metastasis [35].

Overall, we have shown that FABP5 protein expression through our TMA (423 patient samples) and mRNA expression by KM Plot (4,142 patient samples) significantly associates with high grade tumors, TNBC, and disease-free survival. Furthermore, we showed a direct correlation between high FABP5 expression and EGFR expression in breast cancer patients. Using a FABP5^−/−^ mouse model, host FABP5 was found to be important for tumor progression and metastasis. We showed that FABP5 in TNBC tumor cells is important for motility and metastasis. We have also shown for the first time, that FABP5 knockdown leads to decreased EGFR expression, alterations in the EGFR signaling pathways and EGFR-mediated functional affects. In addition, FABP5 was shown to stabilize EGFR protein expression by preventing EGFR ubiquitination and degradation. These studies show that FABP5 could be used as a novel clinical marker for breast cancers, especially TNBC and high grade tumors. Furthermore, our studies indicate an important and novel role for FABP5 in the host and the tumor cell and that targeting FABP5 may prove a viable target to attack EGFR-enhanced TNBC metastasis.

## MATERIALS AND METHODS

### Antibodies and reagents

FABP5 targeted shRNA (Origene) sequences were used as follows:

GCCACAGTTCAGCAGCTGGAAGGAAGATG,
CTGGTGGACAGCAAAGGCTTTGATGAATA,
AGTTTGAAGAAACCACAGCTGATGGCAGA,
GAACAATGTCACCTGTACTCGGATCTATG.

FABP5 cDNA plasmid (Origene) was used to clone FABP5 into pIRES2 vector. FABP5 antibody was purchased from R&D Systems. EGFR/pEGFR (Y1173), GAPDH, and Ubiquitin were purchased from Santa Cruz. pEGFR (Y992, Y1045, Y1068) and Pyk2/pPyk2 were purchased from Cell Signaling Technologies. Recombinant human EGF was purchased from Peprotech. Protein A/Protein G beads were purchased from Calbiochem. MG-132 was purchased from Cayman Chemical. Matrigle was purchased from BD Biosciences.

### Cell lines

Triple negative breast cancer cell lines, MDA-MB-231 and SUM159PT were purchased from ATCC. MDA-MB-436, T47D and MCF7 were provided by Dr. Sharmila Majumdar. MCF10A were provided my Dr. Tsonwin Hai. E0771 cells were obtained from CH3 Biosystems, LLC (Amherst, NY). MDA-MB-231, SUM159PT, T47D, MDA-MB-436, MCF7 and E0771 were maintained in DMEM supplemented with 10% fetal bovine serum and antibiotics. MCF10A cell line was maintained in DMEM/F12 supplemented with 5% horse serum, EGF (20 ng/mL), insulin (10 μg/mL), hydrocortisone (0.5 mg/mL), cholera toxin (10 μg/mL), 5 units/mL penicillin, and 5 mg/mL streptomycin. PyMT cell line was maintained in DMEM/F12 supplemented with 10% fetal bovine serum and 5 units/mL penicillin, and 5 mg/mL streptomycin.

### Mice

Fatty acid binding protein 5 knockout mice in C57Bl/6 background were obtained from Dr. Jill Suttles lab [3, 36]. Mice were housed and maintained at The Ohio State university animal facility under IACUC rules and regulations. Six to eight week old FABP5 knockout mice and C57Bl/6 mice were orthotopically injected into the 4th mammary fat pad with 1×10^6^ PyMT cells suspended in 50:50 Matrigel and PBS. E0771 cells were injected (50,000 cells) into the mammary fat pad, and tumor volume was monitored as described [37]. Mice were sacrificed and primary tumor and lungs were removed for analysis.

### SFBCAS tissue microarray

Institutional review board (IRB) of The Ohio State University has approved the protocol for the constructed SFBCAS tissue microarray (n=423). The construction of the SFBCAS Tissue Microarray and the clinical and pathological characteristics are previously described [38]. Briefly, 824 breast cancer cases prior to 1998 were selected from the National Comprehensive Cancer Network breast cancer database out of 4000 patients. Invasive breast cancer cases were selected based on criteria including stage, grade, histological type, and survival outcomes were available. Pathologists (CM and REJ) reviewed the paraffin-embedded tissue blocks. 564 tissues were acceptable for the TMA containing tumor only (303), tumor and metastasis (226), and metastasis only (35). Data including biochemical markers were available on 423 breast cancer samples. 50 assorted control tissues were placed with the cores (0.6 mm) in quadruplicate blocks. TMA staining was graded as perviously described [39]. Stained samples were dichotomized as low FABP5 expression (staining score of 0 and 1) or high FABP5 expression (staining score of 2 and 3).

### Immunohistochemical analysis

Mouse tumors and lungs were fixed in 4% paraformaldehyde and embedded in parafin for sections [37]. Standard IHC protocols were used per manufacturer's protocol (Vector Laboratories) against FABP5 (R & D Systems, 1: 100) for 60 minutes at room temperature. Detection of bound antibodies was achieved using Vectastain Elite ABC reagents (Vector Laboratories) containing avidin DH:biotinylated horseradish peroxidase H complex with 3,3′-diaminobenzidine (Polysciences) and Mayer's hematoxylin (Fisher Scientific).

### Western blot

Western blotting technique was performed as previously described [37]. Briefly, 50 μg cell lysate was run on a gradient polyacrylimide gel (Invitrogen). Selected proteins were blotted for using primary antibodies (1:1000) and secondary antibodies (1:5000; GE). HRP secondary antibodies were incubated with ECL (Thermo, Sigma) and exposed to film (Bioexpress). Western blot bands were analyzed using ImageJ Software.

### Cell adhesion assay

96-well tissue culture plate was coated with 50 μL Fibronectin (20 μg/mL) overnight in 4°C. Following washing with cold PBS, plates were blocked (0.5% BSA in medium) for 1 hour in a 37°C incubator. Plates were washed (0.1% BSA in medium) and the plates were chilled on ice. 20,000 cells were added to each well. Cells were incubated from 10-30 minutes in a 37°C incubator. On ice, wells were washed with washing buffer four times. Cells were fixed with 2% paraformaldehyde and incubated at room temperature for 10-15 minutes. Wells were washed with washing buffer and stained with crystal violet (5 mg/mL in 2% Ethanol) for 10 minutes. Cells were washed with water and allowed to dry. Five representative pictures were taken at 10x (Zeiess) and attached cells were quantitated.

### Wound healing assay

Cells were seeded for confluency in a 6-well plate. Cells were serum starved overnight. A wound was created using a 200 μL pipette tip. Cells were washed to remove debris and DMEM containing 5% FBS or 100 ng/mL EGF was added. Images were captured at regular intervals and percent wound healing was measured.

### Cell invasion/migration assay

Cell invasion and migration assays were performed as previously described [40]. Briefly, 100,00 cells were seeded into the upper chamber of pre-coated Matrigel invasion chambers (BD Falcon). Cells were allowed to invade in the presence of 5% FBS in DMEM for 24 hours. Cells were stained and quantified as previously described.

### Gelatin zymography

Gelatin zymography was performed as previously described [40]. Briefly, cell supernatants were concentrated using 3,000 kDa filters (Millipore). Samples were run on a 10% gelatin zymography gel and run and developed per manufacturer's protocol (Invitrogen). Gel was visualized by staining SimplyBlue™ safestain (Invitrogen) and image was taken using a Bio-Rad ChemiDoc™.

### Proteasomal degradation assay

Cells were treated with 2, 5, 10, 15, and 20 μM MG-132 or DMSO (veh) for 4 hours in complete medium. Protein was harvested from the cells and western blot was performed as described.

### Ubiquitination assay

Cells were treated with 15 μM MG-132 or DMSO (veh) for 4 hours. Cells were lysed with NP-40 Buffer (150 mM NaCl, 1.0% NP-40, 50 mM Tris, pH 8.0, protease and phosphatase inhibitors (Roche) and gently agitated for 45 minutes at 4 °C. 1 mg of protein was incubated with 5 μg EGFR antibody and antibody control overnight at 4 °C with gentle agitation. 70 μL Protein A/Protein G beads were added to the lysate/antibody mix and gently agitated overnight. Beads were collected by centrifugation for 5 mins at 3,000 RPM and washed with NP-40 buffer three times. Beads were boiled with 2x Laemeli's buffer and loaded with 5% input on a SDS-PAGE gels and western blotting was completed as previously described. An antibody against ubiquitin was used to assay ubqiquitination of the samples.

### Flow cytometry

Cells were seeded and serum-starved overnight. Cells were stimulated with EGF (100 ng/mL) for 15 minutes. Cells were detached from the plate using 1 mM EDTA and agitation for 10-15 minutes. Detachment was observed under microscope. Cells were strained using a cell strainer and fixed with 2% paraformaldehyde in PBS. Cells were washed with PBS on ice and permeabilized using the Fix/Perm kit per manufacturers protocol (BD Biosciences). Cells were then stained with a primary antibody (1:25) for 1 hour on ice. Cells were washed and stained with secondary antibody (1:50) for 1 hour in the dark on ice. Cells were washed and strained through a cell strainer. Cells were submitted to flow cytometry for analysis of EGFR.

### Confocal microscopy

Cells were seeded and serum starved overnight. EGF stimulation was (100 ng/mL) performed for 15 minutes. Cells were then permeablizied and fixed using the Permeabilization and Fixation kit per manufactuors protocol (BD Falcon). Cells were then blocked in 3% BSA in PBS for two hours and room temperature in a moist chamber. Primary antibody (1:100, in 3% BSA) was added to the coverslips and incubated for 1-2 hours at room temperature in a moist chamber. Cells were washed three times with PBS, five minutes per wash. Secondary antibody (1:1000, in 3% BSA) was added to the coverslips and incubated for 1 hour at room temperature in a moist chamber. Cells were washed and mounted using Vectashield. Slides were imaged on a Zeiss Confocal.

### Statistical analysis

Data in the figures are represented as mean ± standard deviation. For the association study between FABP5 expression and EGFR, tumor grade, triple negative, and other biomarkers, chi-square tests or Cochran Armitage trend tests were used. For the disease free survival (DFS) Log-rank tests were used and the results were displayed using Kaplan-Meier survival curves. For other two group comparisons, two sample t-tests or paired t-tests were applied depending on whether the data are independent or correlated. For the tumor volume study, linear mixed models were used to take account of the correlations among observations from the same animal. A *P*-value of *P* < 0.05 was considered to be statistically significant. For all graphs, *P < 0.05, **P < 0.01, ***P < 0.001.

## SUPPLEMENTARY MATERIAL FIGURES



## Abbreviations

FABP5: fatty acid binding protein 5
EGFR: epidermal growth factor receptor
TNBC: triple negative breast cancer
TMA: tissue microarray
MMP: matrix metalloproteinase
ER: esterogen receptor
PR: progesterone receptor
HER2/neu/ErbB-2: human epidermal growth factor receptor 2
