# Supplementary material for: Fatty acid binding protein 5 promotes metastatic potential of triple negative breast cancer cells through enhancing epidermal growth factor receptor stability
Source: Oncotarget. 2015 Jan 31;6(8):6373–85. doi: 10.18632/oncotarget.3442 (PMC4467443; doi:10.18632/oncotarget.3442)
Supplement: Supplementary file 1 [file oncotarget-06-6373-s001.pdf]

Fatty acid binding protein 5 promotes metastatic potential of triple negative breast cancer cells through enhancing epidermal growth factor receptor stability

Supplementary Material

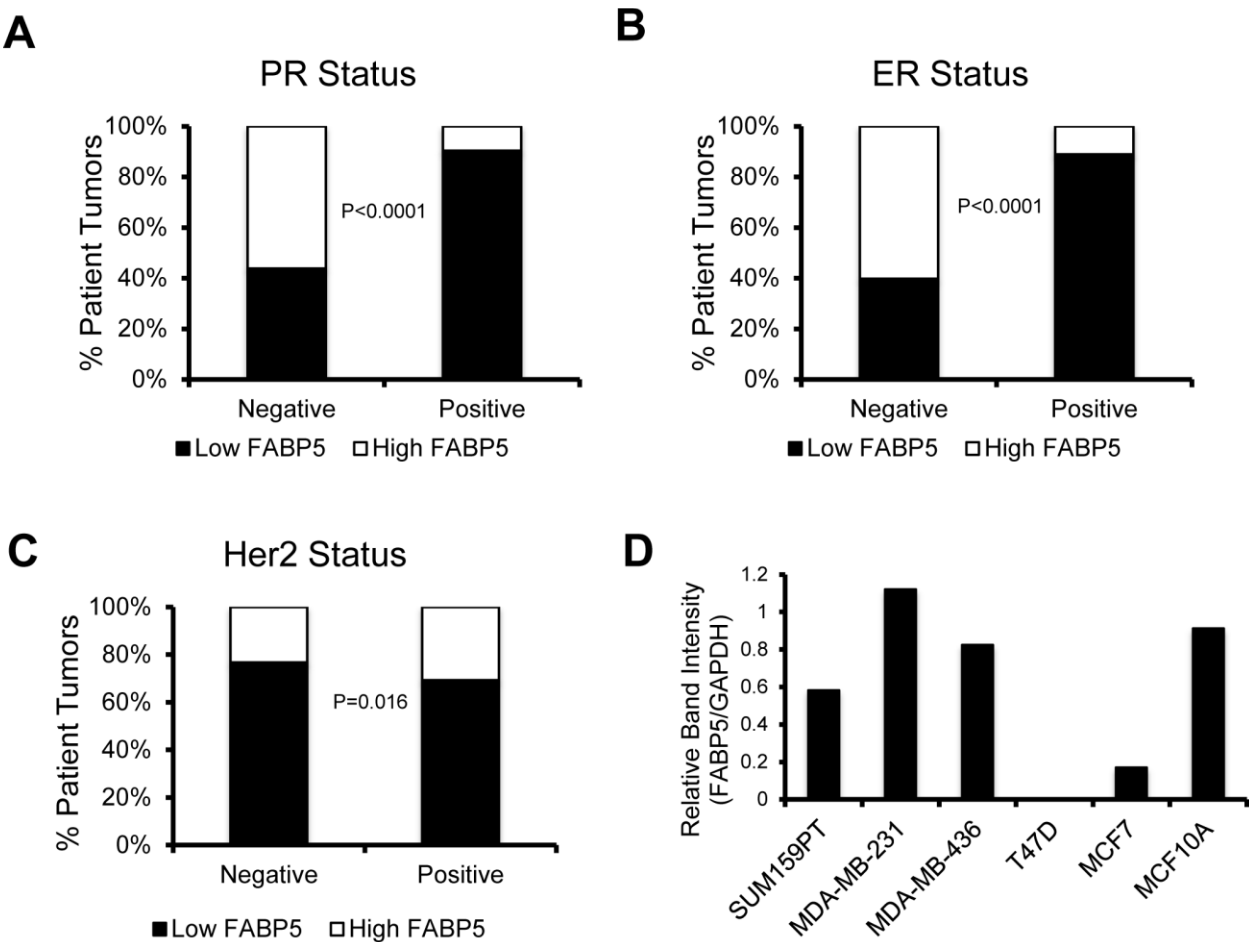

Supplemental Figure S1 : FABP5 expression correlates with individual receptor status in patient data. Graphical representation of FABP5 expression in patient samples compared to PR (**A**, P<0.0001), ER (**B**, P<0.0001), and HER2 (**C**, P<0.016) expression, respectively. **(D)** Quantification of relative FABP5 expression in SUM159PT, MDA-MB-231, MDA-MB-436, T47D, MCF7, and MCF10A cells calculated with respect to GAPDH.

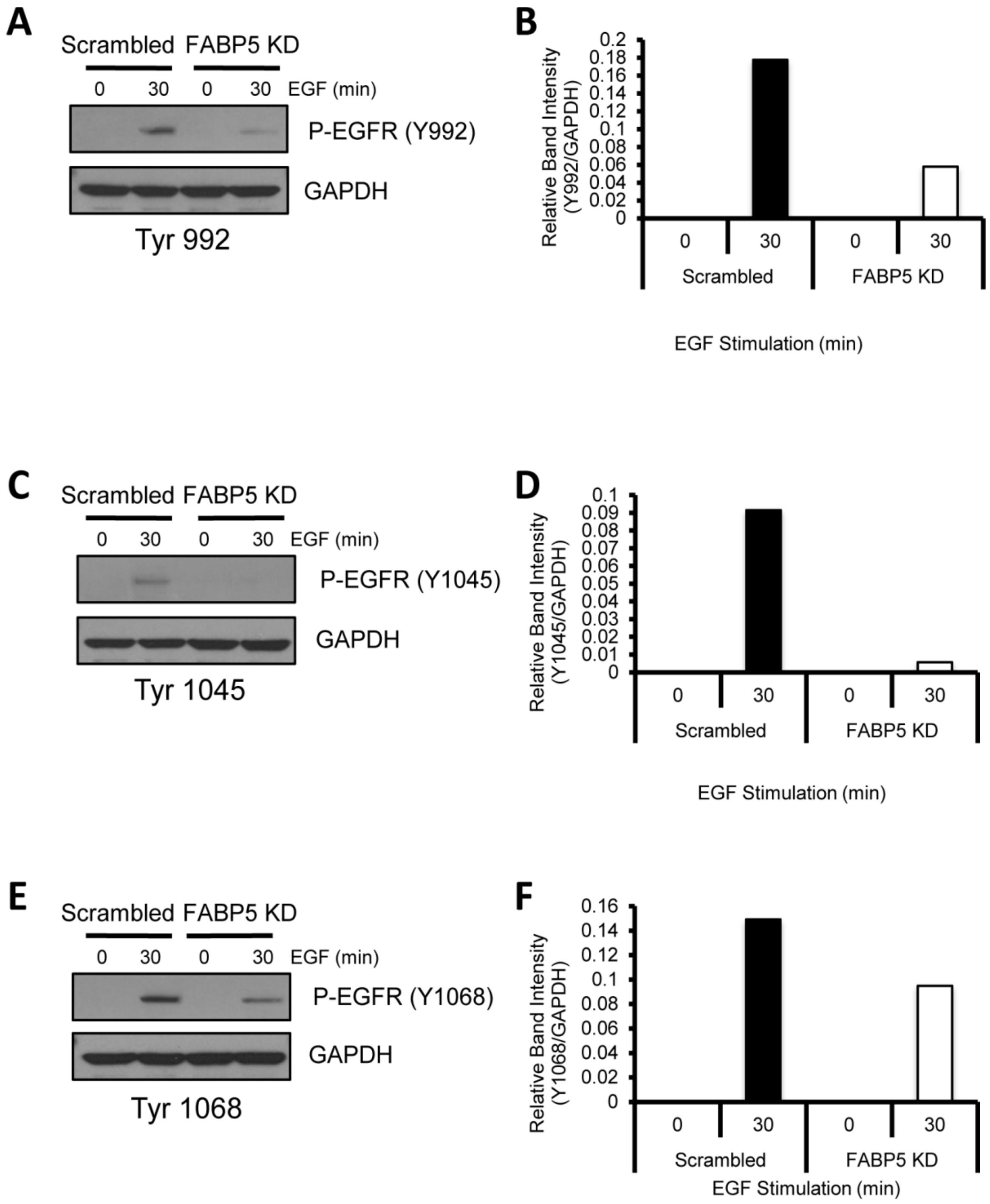

Supplemental Figure S2: Phosphorylation of EGFR decreased in FABP5 KD cells. Scrambled and FABP5 KD cells were serum starved overnight and stimulated with EGF (50 ng/mL) for indicated time points. Cell lysates were harvested and subjected to western blot analysis for **(A&B)** phospho-EGFR (Y992), **(C&D)** phospho-EGFR (Y1045), and **(E&F)** phospho-EGFR (Y1068) with GAPDH as a loading control. Quantification of relative phospho-EGFR expression in scrambled and FABP5 KD cells with varying timepoints of EGF treatments caluclated with respect to GAPDH.
